# Diagnosis of Paediatric Obstructive Sleep-Disordered Breathing beyond Polysomnography

**DOI:** 10.3390/children10081331

**Published:** 2023-08-01

**Authors:** Melissa Borrelli, Adele Corcione, Chiara Cimbalo, Anna Annunziata, Simona Basilicata, Giuseppe Fiorentino, Francesca Santamaria

**Affiliations:** 1 Department of Translational Medical Sciences, Paediatric Pulmonology, Federico II University, 80131 Naples, Italy; ade.corcione@gmail.com (A.C.); cimbalo.chiara93@gmail.com (C.C.); simonabasilicata@gmail.com (S.B.); santamar@unina.it (F.S.); 2 Department of Intensive Cure, Unit of Respiratory Pathophysiology, Monaldi Hospital, 80131 Naples, Italy; annaannunziata@gmail.com (A.A.); giuseppefiorentino1@gmail.com (G.F.)

**Keywords:** sleep disordered breathing, obstructive sleep apnoea, polygraphy, overnight polysomnography, home testing, questionnaire, children

## Abstract

Obstructive sleep-disordered breathing (SDB) has significant impacts on health, and therefore, a timely and accurate diagnosis is crucial for effective management and intervention. This narrative review provides an overview of the current approaches utilised in the diagnosis of SDB in children. Diagnostic methods for SDB in children involve a combination of clinical assessment, medical history evaluation, questionnaires, and objective measurements. Polysomnography (PSG) is the diagnostic gold standard. It records activity of brain and tibial and submental muscles, heart rhythm, eye movements, oximetry, oronasal airflow, abdominal and chest movements, body position. Despite its accuracy, it is a time-consuming and expensive tool. Respiratory polygraphy instead monitors cardiorespiratory function without simultaneously assessing sleep and wakefulness; it is more affordable than PSG, but few paediatric studies compare these techniques and there is optional recommendation in children. Nocturnal oximetry is a simple and accessible exam that has high predictive value only for children at high risk. The daytime nap PSG, despite the advantage of shorter duration and lower costs, is not accurate for predicting SDB. Few paediatric data support the use of home testing during sleep. Finally, laboratory biomarkers and radiological findings are potentially useful hallmarks of SDB, but further investigations are needed to standardise their use in clinical practice.

## 1. Introduction 

Obstructive sleep-disordered breathing (SDB) is a spectrum of different clinical conditions distinguished by upper-airway dysfunction during sleep with snoring and/or increased respiratory effort due to increased upper airway resistance and pharyngeal collapsibility [1]. Obstructive SDB, ranging from primary snoring to obstructive sleep apnoea syndrome (OSAS), has variable severity of intermittent upper airway obstruction that leads to both sleep fragmentation and changes in gas exchange with night-time symptoms, daytime symptoms, and long-term deleterious health effects [2,3,4]. For this reason, it has been assumed that early recognition of SDB and its timely resolution through adequate treatment are necessary to reverse or prevent consequences such as impaired cognitive development, behavioural problems, poor academic performance, increased risk of cardiovascular complications, reduced quality of life, and increased health care use [2,3,4].

SDB occurs in all paediatric ages, from newborn to adolescent, and the prevalence varies depending on the targeted populations studied and the methodology and diagnostic criteria used: the paediatric general prevalence of OSAS is estimated at about 1–5%, with the most predominate age occurring at 2–8 years, while the prevalence of primary snoring is reported to be approximately 7.5%, although it has been reported to be as high as 30% in some published studies [5,6,7,8]. 

Some clinical conditions such as Down’s Syndrome (35% to 70%) [9], Prader–Willi syndrome (93%) [10], cerebral palsy (18%) [11], mitochondrial diseases (11%) [12], obesity (up to 60%) have a higher prevalence [13].

Despite the high prevalence and the need for early recognition of SDB to stem the large spectrum of adverse consequences, the availability and the accessibility of diagnostic services remain a limitation. The American Academy of Sleep Medicine recommends, as part of a routine physical exam, inquiring whether the child or adolescent snores and/or presents signs or symptoms of SDB [14] (see below in Section 3.1). In case of an affirmative answer, the clinicians should refer patients to a physician with expertise in paediatric sleep or to a paediatric sleep laboratory to perform a sleep study. Furthermore, high-risk children (i.e., children with tonsillar and adenoid hypertrophy, craniofacial anomalies, genetic syndrome [15]) or children with comorbidities should be referred to a sleep physician regularly.

Overnight polysomnography (oPSG) is the “gold standard” for the diagnosis of SDB, but it is an expensive and time-consuming tool and can be challenging to undertake in young children. Alternative diagnostic methods should be considered if oPSG is not available [1].

The purpose of this narrative review is to summarise current knowledge about the diagnosis of paediatric obstructive SDB and provide more accurate and up-to-date information about alternative diagnostic tools.

## 2. Polysomnography

Overnight polysomnography (oPSG) is considered the current gold standard method for diagnosis of childhood SDB [1,14]. The oPSG monitors continuously and simultaneously sleep stages and breathing functions allowing one to correlate the sleep architecture information and cortical arousal events with respiratory events. It is performed using the standard recordings of following physiologic functions: brain activity by frontal, central and occipital electroencephalography (EEG) channels, eye movements by bilateral electrooculography (EOG), muscle (tibial and submental) activity by electromyography (EMG), heart rhythm by 2-lead electrocardiography (ECG), peripheral pulse oximetry, oronasal airflow measurement using thermal sensor thermistor and nasal pressure transducer, abdominal and chest wall movements by respiratory inductance plethysmography, and body position by automated detection methods or observation. EEG, EOG, and EMG are required for accurate sleep staging. Video recording is often used to assist in sleep staging [16]. EEG and EMG allow one to record the arousals from sleep associated with SDB in childhood [17]. 

Regarding respiratory events, to identify apnoea/hypopnoea during sleep study in children, the use of an oronasal thermal airflow sensor and/or nasal pressure transducer to monitor airflow is recommended. When these signals are not functioning or not reliable, one of the following alternative apnoea/hypopnoea sensors are suggested: respiratory inductance plethysmography sum (RIPsum), respiratory inductance plethysmography flow (RIPflow), and dual respiratory inductance plethysmography (RIP) belt signals [18]. For monitoring respiratory effort, the Task Force of the American Academy of Sleep Medicine upholds the oesophageal manometry or the calibrated or uncalibrated dual thoracoabdominal RIP belts [18]. Oesophageal pressure measurement is widely considered the very accurate tool for diagnosing the upper airway resistance syndrome (UARS), an SDB characterised by arousals (observed during oPSG) without oxygen desaturations, apnoeas, or hypopneas but with the increase in respiratory effort. The abnormal decrements in oesophageal pressure (Pes) measured which oesophageal manometry reflects intrathoracic pressure and increased inspiratory effort [19]. 

The pulse oximetry with a maximum acceptable signal averaging time of ≤3 s at a heart rate of 80 beats per minutes should be used for detection of oxygen saturation. One of the parameters analysed in oPSG is the Apnoea Hypopnea Index (AHI). It is commonly used to classify OSAS severity: mild OSAS is defined by an AHI score ranging from 1 to 5, moderate OSAS falls within the AHI range of 5 to 10, and finally, severe OSAS is characterized by an AHI score greater than 10 [1].

**Figure 1:**
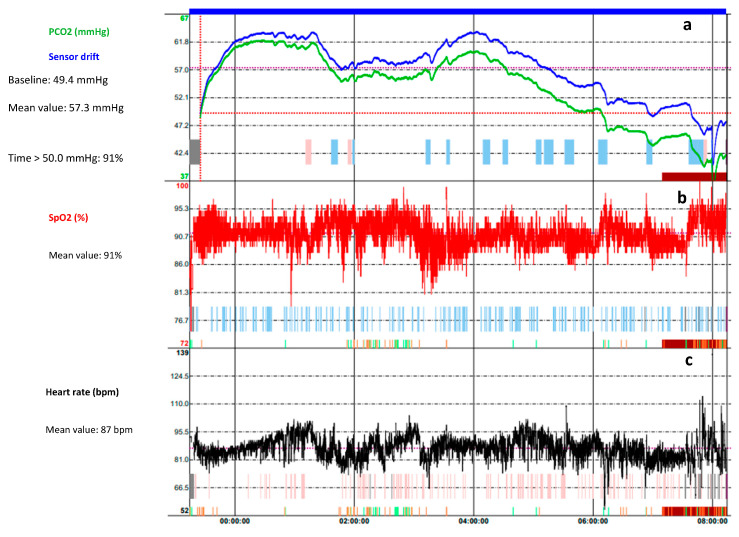
Overnight transcutaneous CO_2_ (TcCO_2_) trend of a 7-year-old female affected by Rett Syndrome. (**a**) The green trace is the measured TcCO_2_ while the blue trace is the drift corrected TcCO_2_; they follow the same pattern over the time. The monitoring shows a hypoventilation pattern (mean TcCO_2_ 57.2 mmHg, time spent with TcCO_2_ > 50 mmHg 91%). The peaks in TcCO_2_ correlated with (**b**) oxygen desaturation events and (**c**) the increase of the heart rate. An improvement in the pattern is observed between 06:00 and 08:00 a.m., as the patient wakes up.

**Figure 2:**
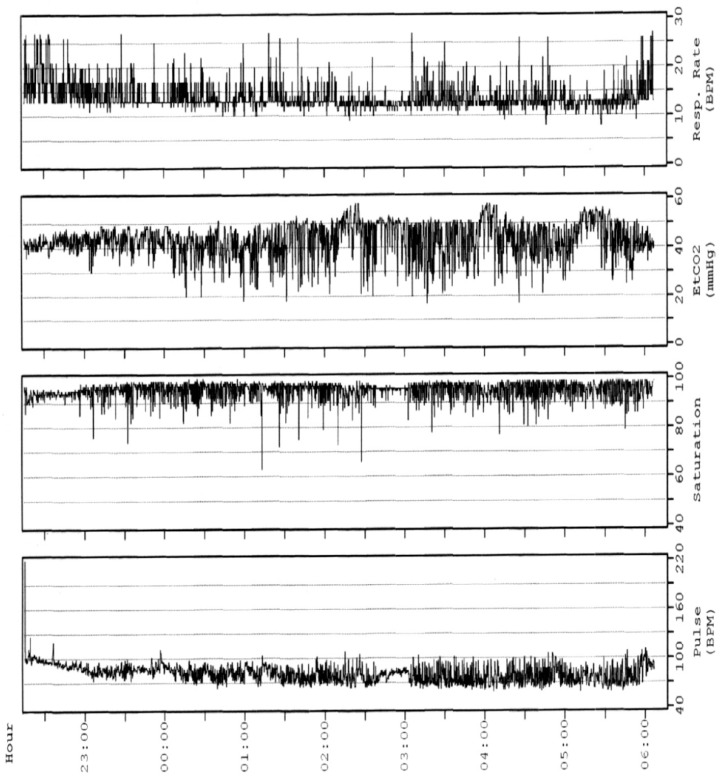
Overnight End-Tidal CO_2_ (P_et_CO_2_) of a 12-year-old male with Duchenne muscular dystrophy. In the initial phase, there is an oxygen saturation without evident desaturation with an P_et_CO_2_ between 40 and 45 between 20 and 22 breaths. After a few hours, the heart rate decreases and phasic oxygen desaturations appear with an ETCO2 that significantly increases with peaks > 50 mmHg while the respiratory rate decreases; such findings are characteristic of sleep apnoea and alveolar hypoventilation.

The oPSG should be carried out by a sleep laboratory with experience in children’s healthcare. Children should be housed in a friendly environment with a parent sleeping next to them. The assessment of oPSG recording requires trained paediatricians and expert technicians to ensure the high quality of recordings, and to assure that the PSG performance, scoring, and interpretation are appropriate for the age and condition of the child [14]. The oPSG is highly costly in terms of human resources, time-consumption, and actual costs. It requires a high expertise equipment with a high staff-to-patient ratio (one to one). A single monitoring can take 8 to 10 h to record and 3 to 4 h to analyse. The determination of the arterial partial pressure of carbon dioxide (PCO_2_) is necessary to the diagnosis of alveolar hypoventilation. Given the difficulty of drawing an arterial sample during sleep, PCO_2_ recording can be performed by means of the end-tidal PCO_2_ (P_ET_CO_2_) or transcutaneous PCO_2_ (P_TC_CO_2_) [18] (Figure 1 and Figure 2). However, the monitoring of PCO_2_ is not performed on a routine basis due to the lack of simple, cheap, and reliable CO_2_ monitors [20]. Unlike for the adult population, paediatric PSG results should not be corroborated over several nights; however, tests should be repeated if results are not conclusive [14]. 

## 3. What Is the Role of Alternative Diagnostic Tools in the Diagnosis of SDB?

### 3.1. Medical History and Physical Examination

Clinical evaluation of paediatric patients with suspected SDB consists in careful evaluation of clinical history and physical examination. These tools alone do not have sufficient specificity and sensitivity to establish a diagnosis of SDB [21]. Based on published evidence, Marcus et al. reported that the positive and predictive value of history and physical examination for the diagnosis of OSAS was 65% and 46%, respectively [14]. However, they are useful to determine whether further evaluation for OSAS is needed and to select patients for instrumental tests. 

Paediatricians should investigate children’s medical history, with a particular focus on prematurity, bronchopulmonary dysplasia, and chronic lung diseases, conditions at risk for SDB [22]. Children with neuromuscular disorders (cerebral palsy, Duchenne muscular dystrophy, and myotonic muscular dystrophy) or genetic syndromes associated with craniofacial anomalies (Down’s syndrome, Beckwith–Wiedemann syndrome, Pierre–Robin sequence) are intrinsically at risk for SDB, due to nocturnal hypoventilation and decreased airway tone, and anatomically determined upper airway obstruction, respectively [23,24,25]. Children with sickle cell disease have both a higher incidence of SDB (19%) [26] and more severe nocturnal desaturation and hypercapnia [27].

As part of a routine check-up, clinicians should inquire about sleep habits. Parents will generally not report sleep problems of their children if not directly questioned. Moreover, in many children, sleep-disordered breathing manifests as hypopneas or obstructive hypoventilation, which may not be easily recognized by parents [28]. A wide range of diurnal and nocturnal symptoms and signs are associated with paediatric SDB. Snoring is the most common presenting complaint reported by parents of children and adolescents with SDB [29]. Other reported paediatric SDB nocturnal symptoms are forced oral breathing, abnormal thoracic–abdominal movements, and witnessed apnoea. Restless sleep with frequent changes of body position, abnormal prone positions, and other sleep positions that minimise obstruction, such as hyperextension of the neck, are also suggestive of children SDB. However, many parents consider it a normal behaviour during sleep in childhood. Further nocturnal symptoms are profuse sweating, which may be related with laboured breathing, and nocturnal secondary enuresis, very pathognomonic for severe OSAS [30]. Night terror and sleepwalking during slow-wave sleep may occur in children and adolescents with a positive family history of parasomnias [31]. Among diurnal symptoms, attention deficit/hyperactivity and irritability are predominant in preadolescent children, whereas excessive daytime sleepiness is a more typical symptom in adolescents or obese children and could be measured by the Epworth Sleepiness Scale [32]. Morning headache, related to carbon dioxide retention, can be reported by children with marked nocturnal hypoventilation [28].

Neurobehavioral sequelae are the result of chronic exposure to intermittent hypoxemia and sleep deprivation [33]. General physical examination includes weight and height growth measurement with careful assessment of growth curves. Obesity is one of the most important risk factors for SDB in both the adult and paediatric populations [34]. The presence of abdominal obesity associated with other signs of insulin resistance, such as acanthosis nigricans, redundant neck tissue, elevated blood pressure, suggests comorbid metabolic disease [35]. On the other hand, SDB could also be associated with a failure to thrive. Although the exact cause is unknown, it may be related to the increased work of breathing during sleep, nocturnal hypoxemia, dysphagia due to enlarged tonsils, a decrease in appetite and alterations in smelling. Abnormal nocturnal growth hormone (GH) secretion pattern and impaired GH action have also been described [36]. A general observation of the child may reveal mouth breathing, adenoidal facies, signs of neuromuscular disease (such as decreased tone), and craniofacial abnormalities associated with a genetic syndrome (midface hypoplasia, choanal atresia, palatal deformities, mandibular hypoplasia) [33]. Inspection of the lateral facial profile is helpful to evaluate for retrognathia, micrognathia, or midfacial hypoplasia, which may contribute to obstructive SDB. The physical examination should include an accurate assessment of the upper airway by an Ear, Nose, and Throat (ENT) specialist and of the extra and intra-oral features by a paediatric orthodontist to identify factors that may cause airway obstruction [9,34,37]. ENT examination should rule out the presence of palatine tonsils hypertrophy [38], chronic rhinosinusitis [39], nasal turbinate hypertrophy, and nasal septum deviation. Friedman classification is a useful tool for grading the oropharyngeal lumen size. It is based on visualization of structures in the mouth with the mouth open widely without protrusion of the tongue: grade 1, both uvula and tonsils are entirely visible; grade 2, uvula is visible but not the tonsils; grade 3, soft palate is visible but not the uvula; grade 4, only a hard palate is visible [40]. The adenoid tonsil hypertrophy is assessed via nasal fiberoptic flexible endoscopy, graded according to Cassano [41]. 

Dental examination should evaluate skeletal class (retrognathic, orthognathic, or prognathic), molar relationship (classified on both the left and right sides, according to Angle’s classification as Class I, II, or III), presence of overbite and posterior cross-bite [9,37] and narrow palate (intermolar distance, defined as the distance between the palatal grooves of the upper first molars with the gingiva) [42].

To identify an altered lingual frenulum and relate anatomical frenulum alterations to the oral functional alterations, a protocol that evaluates history and clinical examination was proposed by Marchesan [43]. The clinical examination includes a first part that consists in four general tests and the second one in four functional tests.

Clinicians should also assess malformations of the sternum and thoracic cage that may predispose the patients to obstructive hypoventilation, and the presence of clubbing of the fingers, related to chronic lung disease with hypoxemia and hypoventilation [28]. 

High blood pressure and eventual signs of pulmonary hypertension, as a loud second pulmonary heart sound, should be included in the clinical evaluation since they are manifestations of severe disease [44].

### 3.2. Questionnaires

Several questionnaires have been developed to identify the clinical history of paediatric patients with suspected SDB. Their diagnostic accuracy is too low to be considered as alternative diagnostic method to PSG; however, they are useful screening tools in the diagnostic process of paediatric SDB.

The Paediatric Sleep Questionnaire proposed by Chervin et al. [45], validated in several languages, is the most widely used questionnaire. It consists of 22 items focusing on snoring, excessive daytime sleepiness, and behavioural problems. PSQ has a sensitivity of 0.85 and specificity of 0.87 (cutoff 0.33) in otherwise healthy children aged 2–18 years for identifying SDB confirmed by polysomnography. In a systematic review by De Luca Canto et al., about diagnostic capability of questionnaires and physical examinations for diagnosing SDB in children, only the Paediatric Sleep Questionnaire (PSQ) showed a diagnostic accuracy good enough to be used as a screening method for SDB [46]. 

The current guidelines of the European Respiratory Society Task Force define PSQ as a useful instrument for identifying children with an obstructive apnoea–hypopnoea index (AHI) > 5/h, detecting OSAS-related neurobehavioural morbidity and assessment of its improvement after adenotonsillectomy [1]. However, PSQ is not a good screening tool for OSAS in children with complex underlying disorders, e.g., neuromuscular disorders, cranio-facial anomalies, and Down’s syndrome [47]. Other questionnaires have been proposed for paediatric SDB. Among these, the first questionnaire described in the literature is the Brouillette score [48], which combines three sleep-related symptoms: snoring, observed apnoeas, and difficulty breathing. 

The OSA-18, a 7-category scale with 18 questions, has been developed to evaluate the impact of OSAS on health-related quality of life [49]. Both have shown a poor sensitivity and specificity for predicting PSG findings.

Owens and colleagues developed the 33-item children’s sleep habits questionnaire (CSHQ), validated in several languages [50], which yields scores on eight sleep domains, including sleep-disordered breathing. A cut-off score of 41 has been shown to accurately identify paediatric sleep disorders with sensitivity and specificity of 0.80 and 0.72, respectively.

The STOP-Bang questionnaire is widely used in the adult population to predict the risk of OSAS and the need for PSG, also existing in a modified version for adolescents (teen STOP-Bang). The following parameters are considered: snoring, tiredness, observed apnoea, blood pressure ≥ 95th percentile, body mass index > 95th percentile, academic problems, neck circumference > 95th percentile for age and male gender. The teen STOP-Bang questionnaire has high negative predictive value for OSAS, and it could be useful for identifying the subgroup of teenagers at high risk of OSAS who should be referred for PSG with priority [51]. 

The sleep clinical record (SCR), proposed by Villa et al., is a useful screening tool for paediatric SDB that combines clinical history with physical examination findings [52]. It is a validated instrument that consists of three main items. The first concerns physical examination of the nose, oropharynx, and dental and skeletal occlusion; the second concerns patient history and involves the Brouillette score [48]. The third item assesses the presence of inattention and hyperactivity symptoms using the ADHD rating scale for school-aged children. The SCR score had a sensitivity of 96.05% and a specificity of 67%. Children with a low SCR score have a low risk of severe OSAS. The limitation of the SCR is that it was validated in children with suspected SDB and not in asymptomatic children. Moreover, SCR is the most complex and time-consuming screening questionnaire with a required time of about 30–60 min. 

### 3.3. Nocturnal Oximetry

Continuous overnight recording of oxygen saturation, either at home or in the hospital, is an appealing alternative to oPSG due to its cost-effectiveness, its availability in most centres, and its being easily performed and analysable. Obstructive apnoea and hypopnoea may lead to recurrent brief desaturations. Brouillette et al. defined a desaturation as a drop in SpO2 greater than 4%, and a cluster of desaturations as the occurrence of five or more desaturations within a 10 to 30 min period. They demonstrated that a periodic cluster of 3 or more desaturations > 90% on continuous overnight oximetry had a 97% positive predictive value for OSAS in healthy children. The authors concluded that oximetry could be the definitive diagnostic test for straightforward obstructive sleep apnoea attributable to adenotonsillar hypertrophy in children older than 1 year of age without other medical problems and with a history strongly suggestive of OSAS. They could be referred directly for adenotonsillectomy surgery, without proceeding to a more detailed sleep study [53]. However, in children, obstructive events often do not cause significant desaturation, which means they may go undetected by pulse oximetry alone. Additionally, movement artefacts can result in false desaturation events, masking the true depth and frequency of desaturations. Therefore, a negative or inconclusive oximetry result cannot be used to rule out OSAS [48]. Nixon et al. showed that overnight pulse oximetry can be used to estimate the severity of OSAS, to shorten the diagnostic and therapeutic process for children with more severe disease, and to aid clinicians in prioritisation of surgery and scheduling perioperative care [54]. The McGill oximetry score (MOS) was developed as a scoring system to assess the severity of OSAS and prioritise the timing of surgery. Differentiation was based on the severity of desaturations and each score was associated with a recommendation for timing of surgery or for oPSG [54].

### 3.4. Respiratory Polygraphy

**Figure 3:**
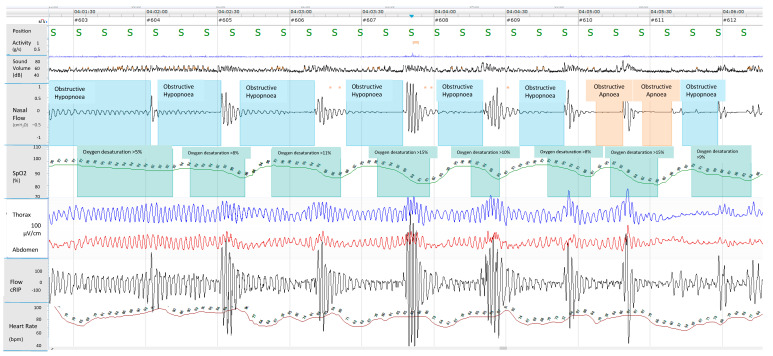
Respiratory polygraphy of a 9-year-old male with obesity. Cardiorespiratory monitoring used sensors to detect body position (position), activity (g/s), sound volume (Sound Volume (dB)), nasal airflow (Nasal Flow (cmH_2_O)), oxygen saturation (SpO2 (%)), chest (Thorax) and abdominal (Abdomen) movements (inductance plethysmography), respiratory inductance plethysmography flow (Flow cRIP), and pulse rate ( Heart Rate (bpm)). The monitoring showed several events of obstructive apnoea and hypopnoea characterized by deep fall of the nasal pressure > 90% and >30%, respectively, increase of respiratory effort with chest and abdominal movements, oxygen desaturation > 3%.

Respiratory polygraphy (RP) is a technique that monitors cardiorespiratory function (i.e., airflow, chest and abdominal wall movements, pulse oximetry, the ECG and position) without simultaneously assessing sleep and wakefulness (Figure 3). Although the use of RP is considered suitable and appropriate under certain conditions in adults [55], there is optional recommendation in children [14]. However, it seems that there is a discrepancy in the use of RP in the diagnosing of paediatric OSAS in different countries. In Europe, in fact, many laboratories perform respiratory polygraphy, unlike the US and Australia [56].

Currently, there are few paediatric studies directly comparing RP and oPSG [56,57,58]. Data showed that RP underestimates the apnoea–hypopnoea index (AHI), firstly, because this monitoring identifies only the hypopneas associated with desaturations, whereas events that just result in an arousal are missed. Furthermore, underestimated AHI obtained by RP is calculated using the total time recorded (TRT) as denominator, which could be greater than the total sleep time (TST) considered in oPSG. However, several published studies on RPs versus PSGs agree that in the case of moderate–severe OSAS, the RP showed a good sensitivity and specificity, and a positive predictive value [56,58,59,60]. Only one paediatric study has examined the clinical management implications of the two different diagnostic approaches. Relying on the therapeutic decision on RP instead of PSG results, there were no differences in therapeutic management for children who do not have OSAS as well for children who have severe OSAS [56].

### 3.5. Nap Studies

One of the solutions to the limited supply of overnight sleep studies is to perform full PSG in the laboratory during a daytime nap. The advantage is the shorter duration and lower costs. In adults with severe OSAS, a daytime PSG compares favourably with overnight studies in terms of detecting and establishing the severity of OSAS. Only two studies compared daytime nap and oPSG in children. Marcus et al. [61] showed that SDB detected by nap PSG are always confirmed by oPSG. However, nap PSG significantly underestimated abnormalities detected by oPSG. This result was confirmed by Saeed et al. [62], which demonstrated that no individual nap study parameter is sensitive enough for the prediction of OSAS. Hence, a negative or inconclusive nap PSG does not exclude the diagnosis of SDB and if the clinical suspicion is high, an oPSG should be performed. The higher severity of SDB during the overnight as compared to the nap studies could be explained by the increased REM sleep that is rarely seen in daytime nap studies. 

The limitations of daytime nap study are that it may not include rapid eye movement (REM) sleep and may be affected by circadian variation in sleep patterns. Moreover, it may require sedation to facilitate sleep, and although chloral hydrate seems a safe medication, its effect on the depression of upper airways muscle tone is not well known in the paediatric population [62]. The use of longer nap studies, which would include REM sleep, could make nap PSG a more useful screening tool.

### 3.6. Home Testing

Ambulatory sleep testing performed in the home, outside the sleep laboratory, with a portable monitoring device, is an attractive alternative to in-laboratory oPSG. These portable devices can provide for the recording of a single channel such as oximetry, or 4 channels, such as oxygen saturation, heart rate, respiratory bands, airflow (hRP), or a comprehensive monitoring (hPSG). 

No sufficient evidence supports the use of home testing in children, unlike in the adult population [63,64]. 

#### 3.6.1. Home Pulse Oximetry

Despite controversial data about the accuracy of at-home pulse oximetry in children to identify OSAS [65], the good repeatability from night to night of the results of monitoring performed in the home is widely demonstrated [66,67]. Thus, when the first pulse oximetry is inconclusive, there is little benefit to routinely performing a second night of pulse oximetry, but the final diagnosis will be made following oPSG. 

Encouraging results have emerged from the monitoring of oximetry combined with pulse rate variability by means of smartphones that have incorporated a pulse oximeter. Due to the extraordinary market penetration of smartphones all around the world, these devices could represent future tools, readily available, for the screening and the diagnosis of SDB [68]. At the same time, a recent study showed that using advanced mathematical algorithms, home pulse oximetry achieves a good accuracy as an alternative diagnostic tool for paediatric OSAS, especially moderate–severe paediatric OSAS, which corresponds to the point of upward inflection in morbidity risks [69]. 

Another home portable monitoring device is the Watch PAT 200 (WP200), it analyses arterial oxygen saturation, heart rate, and peripheral arterial tonometry (PAT) signal and reports the AHI based on the PAT signal. Only one study in adolescents showed a high correlation and good agreement in AHI and Oxygen saturation between the WP200 and PSG [70].

#### 3.6.2. Home RP

Home respiratory polygraphy (hRP) has progressively shown several benefits for the diagnosis of childhood OSAS, to increase the diagnostic accuracy and to simplify the diagnostic process [58,71,72]. AHI measurements obtained from hRP appear not to differ significantly from PSG results, especially in older children. Despite the numerous benefits of this technique, one of the technical disadvantages is obtaining an adequate nasal airflow. This could be particularly harder in younger children and mouth breathers, with reduced nasal airflow [71]. Furthermore, movements during sleep could remove the nasal cannula. These factors could interfere with the correct hRP results. In order to fix these issues, Chiner et al. suggest a minimum period of recording of 3 h, maintaining at least two channels, including SpO2 and band or flow. 

#### 3.6.3. Home PSG

Home PSG (hPSG) appears to be a feasible and technically reliable method, showing no significant differences between the interpretability of hPSG and in-laboratory oPSG. Several studies demonstrated that it is possible to obtain good quality hPSGs recordings, with no significant differences with the in-laboratory ones [59,73,74]. A recent study shows excellent concordance between hPSG and in-laboratory oPSG for diagnosing OSAS in children aged 5–18 years. hPSG could be more representative of a typical night routine for children with the additional benefit of decreasing waiting times for PSG performed in a laboratory, improving access to this diagnostic method and possibly reducing the costs of identifying and treating OSAS [75]. 

#### 3.6.4. Video Recording

Home-made video recordings are sometimes provided by parents of children with suspected sleep apnoea. In an old study of 1996, authors compared the result of a scoring system (based on noisy breathing, movements, walking episodes, apnoea, chest retractions, and mouth breathing) applied to a 30 min home video recording of children during sleep, with PSG findings. They found that the score was highly correlated with PSG results, with high sensitivity and relatively low specificity. These results make the home video-recording a reliable screening test for OSAS and other SDB in children, useful for selecting patients for polysomnography [76]. In an age of mobile screens, with the number of smartphone users at 6.92 billion worldwide, which translates to 86.29% of the world’s population owning a smartphone, Thomas RJ and colleagues [77] proposed and validated a simple clinical tool to study breathing abnormalities, consisting in a short video recording made on a smartphone. By agreement with previous study, they showed that video smartphone scores had both high sensitivity and negative predictive value, but low specificity. A limitation of the video monitoring is that it may not capture rapid eye movement (REM) sleep, with an underestimation of OSAS, which is often REM sleep-related in young children. On the other end, it records only a portion of the night’s sleep, which may not be representative because parents probably select the worst periods for review by a physician.

### 3.7. Sonography

Habitual snoring, a prominent clinical entity of SDB, should already be considered a pathological condition in children associated with various disorders from reduced neurocognitive function with inattentive and hyperactive behaviour [78] to cardiovascular alterations [79]. Several sensors for detecting the snoring are recommended by the American Academy of Sleep Medicine such as the unfiltered nasal pressure signal, piezoelectric sensors to detect vibration, or acoustic sensors (e.g., microphone) to record sound [18].

Sonography consists in analysing snoring sounds during sleep to evaluate breathing. It could be used alone or in combination with other tools such as pulse oximetry, even though its validity is demonstrated only on adults [80]. 

### 3.8. Laboratory Biomarkers

Laboratory biomarkers, from several biological samples, can be very useful to screen or confirm SDB diagnosis. 

Gozal et al. [81] in 2009 quantified 7 urinary proteins from 60 children affected by OSA (diagnosed by polysomnography) and between them identified especially four of these: uromodulin, urocortin-3, orosomucoid-1, and kallikrein. If the urinary concentrations of these four proteins is beyond the calculated cut off for three or more times, this outcome is related to OSA with a sensitivity of 95% and specificity of 100%. A following study confirmed that this combination of urinary proteins was an accurate tool to predict OSA in children [82]. Another potential biomarker is the urinary level of neurotransmitters. An overnight increase in creatinine-adjusted epinephrine and norepinephrine urinary levels were recorded in children with OSAS in association with an increase in urinary GABA levels and a decrease of taurine. The study comprised 50 subjects affected by OSAS and 20 controls [83]. The increase in morning urinary levels of catecholamines compared with the urinary levels of the evening prior to sleep demonstrated autonomic nervous system dysfunction due to hypoxia and hypercapnia overnight in children affected by OSA with heightened sympathetic tone. In 2022, a systematic review and meta-analysis analysed data from 9 studies with the aim of stratifying the catecholamine urinary levels between children affected by sleep-disordered breathing of different grades of severity. Urinary noradrenaline increased in all patients with SDB; instead, subjects with OSA had higher urine levels of noradrenaline and adrenaline. The urinary noradrenaline level is higher in severe OSAS, confirming the autonomic imbalance in children affected by OSA; the authors proposed the possible use of these biomarkers to screen children at risk for SDB and predict the severity also [84].

The products of oxidative stress were also proposed as SDB biomarkers. A study conducted on 65 children affected by SDB demonstrated an increase in urinary levels of 8-isoprostane and a positive linear correlation between its levels and the severity of the disease regardless of the patients’ BMI [85]. The record can be explained by the intermittent hypoxia during sleep in SDB patients that leads to lipid peroxidation and inflammatory activation. A previous work identified high levels of 8-isoprostane also in the breath condensate of children with high clinical scores for OSAS [86]. Among blood biomarkers, several studies reported elevated red cell distribution width in adults affected by sleep breathing disorders and its correlation with the severity of OSAS. The potential mechanism is the inflammation induced by hypoxia that inhibits the bone marrow red cell production with a release in the circle of immature red cells and also the higher red cell turnover due to inflammation status. In children, one study showed that preoperative elevated red cells distribution width is associated with an increased risk of respiratory adverse events in children undergoing adenotonsillectomy for OSAS. This result can help in postoperative and emergency management of these patients [87]. A recent study explored the potential use of EPO levels as a paediatric sleep disorders marker. Data from 90 children were analysed and the study demonstrated a higher level of EPO in children affected by OSAS and its correlation with AHI score. The inconstant O2 tension overnight in OSAS patients activates the hypoxia-inducible factor 2 (HIF-2) that regulates transcription of several genes, including EPO: 2 h of continuous hypoxia as well as 4 h of intermittent hypoxia results in a 50% increase of EPO [88]. Another potential diagnostic tool for children affected by SDB is the exhaled breath profile analysis using the E-nose technology that detects the volatile compounds in exhaled breath. The study compared the exhaled profile collected by lower airways of eighteen children affected by OSAS and ten habitual snoring and demonstrated an altered exhaled pattern in OSAS children, potentially related to lower airways and systemic inflammation [89].

Since the association between OSAS and metabolic alterations is well known, another hallmark to identify children at risk of SDB could be the study of metabolic biomarkers. Bushan et al. demonstrated the association between OSAS severity and higher levels of fasting insulin, HOMA index, and glucose on blood on 76 paediatric patients. The phenomenon could be explained by the OSAS-related hyper-sympathetic activity, hypercortisolism, and inflammation status [90]. A recent work demonstrated also that IL-6, IL-8, IL-17, IL-18, MIF, Hs CRP, TNF-PAI-1, and leptin levels were significantly higher in 190 Asiatic obese patients affected by OSAS, according to the strong inflammatory status of this condition [91]. Further studies are needed to standardise the use of these biomarkers as possible tools to screen paediatric patients at risk of SDB, to identify them and predict the severity of disease. 

### 3.9. Radiologic Studies

Several radiological techniques are available to evaluate the upper airway abnormalities predisposing to obstructive SDB, including lateral neck radiograph, cephalometrics, computerised tomography (CT), magnetic resonance imaging (MRI), and post-processing of these images using computational fluid dynamics (CFD) [92]. Lateral neck X-ray is a simple technique that visualises the upper airway from the nasopharynx to the upper thoracic trachea. It has relatively good predictive values for the diagnosis of OSAS, that can also be improved by incorporating certain clinical factors (obesity, mouth breathing, nocturnal enuresis, observed apnoea during sleep, intrusive naps, and enlarged tonsils) [92]. Cephalometry is a standardised lateral radiographic view of the head which includes the entire skull and soft-tissue structures. Cephalometry determines areas of narrowing and possible obstruction, but there is no evidence about its sensitivity or specificity. Limitations of radiological techniques are the radiation exposure of children, the 2-dimensional nature, and the low resolution that does not allow for a clear visualisation of soft tissue abnormalities. Furthermore, since the radiograph is taken while the patient is awake, in an upright position, it does not adequately demonstrate dynamic airway collapse occurring during sleep.

Both Computed Tomography (CT) and Magnetic Resonance Imaging (MRI) can help providing a detailed anatomical estimation of the upper airways and surrounding tissues. 

MRI is more frequently used than CT because of the amount of radiation produced by CT. On the other hand, MRI is noisy, has longer acquisition times, and sedation or anaesthesia to tolerate the long time is needed for the examination. A possible side effect of sedation is a collapse of upper airways, not present in normal conditions. 

Cine-MRI captures dynamic changes of the upper airway throughout the respiratory cycle. It has been applied in children with persistent OSAS following adenotonsillectomy or for high-risk children with multiple sites of upper airway obstruction, especially in those with complex conditions [93,94]. Computational fluid dynamics (CFD) can help in predicting the internal flow dynamics of upper airways, based on MRI or CT findings. Even though the use this technique is not spread, there is a correlation between CFD parameters and OSAS severity [92]. 

Several studies involving MRI or CT have shown a correlation between anatomical features of upper airway, such as tonsils and adenoids size, soft palate, retropalatal region, and OSAS severity [95,96,97]. However, no data on the sensitivity or specificity of these techniques have been published. Drug-induced sleep endoscopy (DISE) enables dynamic evaluation of the airway with a flexible endoscope while the child is in a pharmacologically induced sleep-like state [98]. This method allows the identification of areas of obstruction which may become surgical targets. It is particularly useful in patients with post-adenotonsillectomy persistent OSAS [28] or those considered to be at high risk for residual OSAS after surgery (patients with severe OSAS, obesity, Down’s syndrome, craniofacial syndromes, or neuromuscular disorders). Despite its increasingly widespread use, larger and more rigorous studies should be performed in order to evaluate outcomes associated with DISE-directed surgical management and identify subpopulations who benefit most from this approach.

## 4. Discussion

SDB are common in children. In this population, pathophysiology, clinical manifestations, diagnosis, and management of SDB are different than in adults. It is widely accepted that children and adolescents with suggestive history and/or with conditions or morbidity at risk for SDB should perform a sleep study [1,14]. This is recommended also in children with suspected OSAS prior to adenotonsillectomy, especially in the presence of factors predicting long-term persistence of OSAS after surgery or when the need for treatment is unclear [1]. The oPSG, conducted in sleep laboratories, remains the gold standard for the diagnosis of SDB; thus, the diagnosis is critically dependent on the availability and accessibility of oPSG. Many children with significant SDB may remain undiagnosed for several reasons: first of all, there are few dedicated paediatric sleep laboratories worldwide. Performing and evaluating paediatric oPSG requires experience and expertise that are part of the training and certification of sleep specialists. Currently, there is a relative lack of specialists with the appropriate expertise in paediatric sleep disorders [99], increasing waiting lists. Secondly, oPSG is expensive as it requires access to an overnight bed in the hospital and the presence of a sleep technologist during the night, resulting in being very time-consuming, and causing discomfort for the child and his family. Many of these disadvantages could be overcome with hPSG, which can be performed successfully in term of cost savings, of diagnostic accuracy, and of high success rates of recording, especially when conducted by trained staff [100,101]. Overall, data about other home testing are conflicting [58,65,71,102]. Nocturnal Oximetry is certainly accessible, inexpensive, and relatively quick and easy to analyse [53]. It detects moderate and severe OSAS with satisfactory diagnostic performance [103]. Given the high positive predictive value (close to 100%) if the oximetry recording is positive (McGill score greater than 1), performance of polysomnography does not appear to be required to refer the patient for adenotonsillectomy. On the contrary, a normal nocturnal oximetry does not rule out OSAS and a PSG is required for diagnosis [103]. The diagnostic accuracy of the nocturnal oximetry could be improved by the combination of the nocturnal recording with the clinical data, such as SCR [104] or questionnaires [105,106]. In addition, some studies showed preliminary exciting data about the combination with heart parameters [68,70]; nevertheless, additional feasibility studies are needed to confirm them.

Several studies proved that RP underestimates AHI compared to oPSG [56,57,58]. This is mainly due to the loss of respiratory events associated with arousals rather than desaturation, and to the calculation of AHI using as denominator the TRT. In laboratory RP, an accurate estimate of actual sleep time can be obtained by the presence of a sleep technician and concurrent video monitoring, although some authors argue that the total sleep time based on video is less accurate than when determined by EEG [56]. There are significant differences between home and in-laboratory RP because hRP does not include video and the presence of trained staff. The quality of recordings, especially in young children, depends on the set-up of the equipment and adjustments of any sensors during night. Despite these factors, hRP could be convenient in the matter of lowering performance costs and of reducing waiting lists and in-lab discomfort for children and families.

Laboratory biomarkers are a promising challenge to improve the diagnosis of OSAS in children, potentially leading to more efficient and comfortable diagnostic methods for patients and their families, even though their use is not standardised at the moment. Further studies are needed to validate these biomarkers, refine their cutoff values, and determine their real utility in routine clinical practice.

Radiological techniques are especially used for research purposes to evaluate the pathophysiology of OSAS. In clinical practice, they are typically reserved for upper airway evaluation in children with comorbidities such as craniofacial anomalies or neurological conditions. These methods may assist in identification of level of obstruction and treatment selection. 

Further studies are needed to identify the role of upper airway imaging in diagnosing OSAS, as minimal data have currently been published regarding the sensitivity and specificity of each test. 

Finally, in settings where the access to oPSG is unavailable or with long waiting lists, alternative tools for the diagnosis of SDB can be applied in children with a high probability of apnoea–hypopnoea based on clinical evaluation. These methods should not be performed in children with complex chronic diseases, such as neuromuscular disease, underlying lung disease, or with evidence of significant comorbidities, such as obesity. These patients have a greater risk for other sleep-related phenomena, often erroneously attributed to apnoea–hypopnoea, and for nocturnal hypoventilation; in the aforementioned cases, oPSG and CO_2_ monitoring are necessary. The combination of two or more alternative tools could enable more accurate estimates of SDB, improving the sensibility compared to the tool performed alone. When using alternative tools, positive results are helpful for confirming the diagnosis of SDB; on the other hand, a negative result does not rule out SDB; thus, further investigation, namely, oPSG, is mandatory. Finding a paediatric diagnostic tool with the right balance between diagnostic accuracy and minimising cost could represent the next challenge.

**Figure 4:**
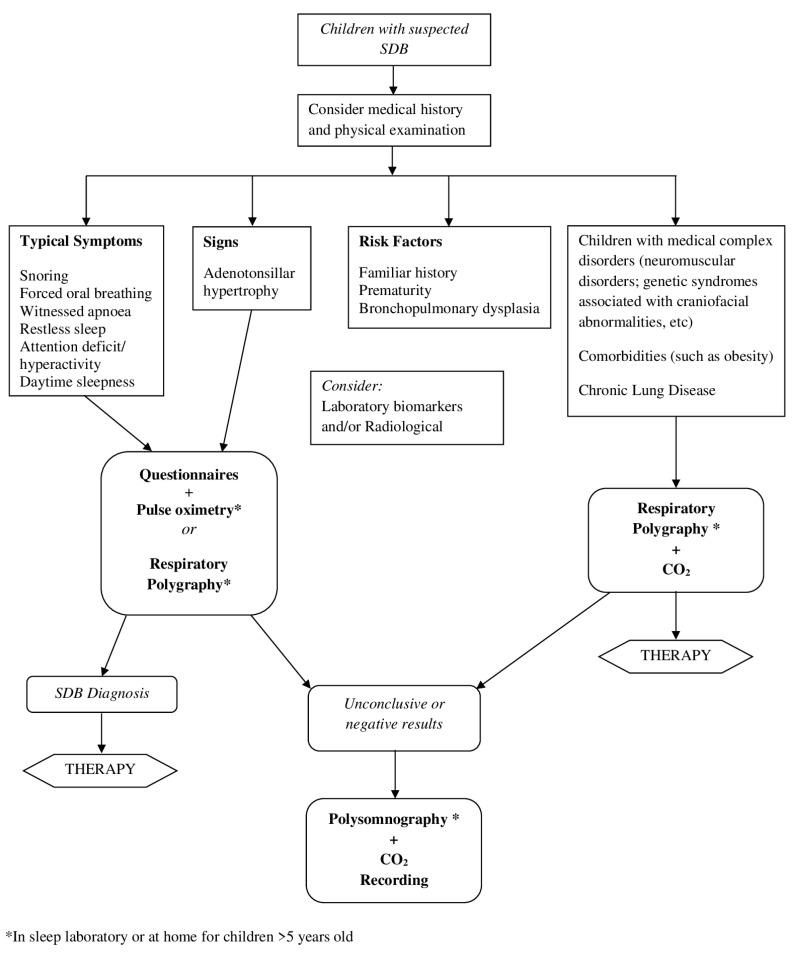
Proposal of algorithm for management of children with suspected SDB.

We propose a synthetic algorithm for management of children with suspected SDB (Figure 4). 

## Data Availability

Data is contained within the article.
